# Successful treatment of posttraumatic phlegmasia cerulea dolens by reconstructing the external iliac vein: a case report

**DOI:** 10.1186/1752-1947-8-149

**Published:** 2014-05-14

**Authors:** Haidi Hu, Yongchang Cai, Chuanjiang Wang, Chunqing Yang, Zhiquan Duan, Jian Zhang, Shijie Xin

**Affiliations:** 1 Division of Vascular and Thyroid Surgery, Department of Surgery, The First Affiliated Hospital, China Medical University, Shenyang 110001, China

**Keywords:** Phlegmasia cerulea dolens, Posttraumatic, Reconstruction, Trauma, Iliac vein

## Abstract

**Introduction:**

Phlegmasia cerulea dolens is a rare condition caused by complete venous occlusion leading to impaired arterial flow. To prevent progression to limb gangrene, prompt diagnosis and treatment initiation are paramount. Here we report a rare case of posttraumatic phlegmasia cerulea dolens after ligation of the iliac vein to save the patient's life, with successful treatment by reconstructing the external iliac vein. This is the first report of posttraumatic phlegmasia cerulea dolens induced by iliac vein ligation.

**Case presentation:**

A 49-year-old Chinese man was admitted to a local hospital for severe knife trauma with massive intraperitoneal bleeding. During exploratory laparotomy, he was diagnosed with traumatic rupture of his left external iliac vein without injury to the iliac artery. The proximal and distal parts of his injured external iliac vein were ligated to control the bleeding and rescue him, but his left leg quickly became severe swollen, cyanotic and pulseless. He was diagnosed with posttraumatic phlegmasia cerulea dolens after being transferred to our university hospital. After a retrievable filter was placed in his inferior vena cava via his right femoral vein, he underwent reopening of his abdomen followed by successful surgical reconstruction of his left iliac vein. He was treated with anticoagulation therapy postoperatively and his signs and symptoms improved markedly. He was discharged in a stable condition, with nearly full resolution of symptoms, 35 days after the operation.

**Conclusions:**

Our case demonstrates that ligation of an injured iliac vein may induce phlegmasia cerulea dolens in a posttraumatic scenario; prompt reconstruction of the iliac vein to restore the venous drainage is an effective treatment for phlegmasia cerulea dolens with impending gangrene.

## Introduction

Phlegmasia cerulea dolens (PCD) is a rare condition caused by complete venous occlusion that leads to impaired arterial flow [1-3]. In the most cases, PCD presents as the result of an extreme form of deep venous thrombosis with proximal localization of the blockage, most frequently in the ileofemoral area; however, there are a few reports of PCD resulting from trauma [1,4]. PCD continues to have mortality rates of 25% to 40% and amputation rates of 20% to 50%, respectively [2,3,5]. To prevent progression to limb gangrene, prompt diagnosis and treatment initiation are paramount. Here we report a case of posttraumatic PCD successfully treated by reconstructing the injured external iliac vein and limb loss was avoided.

## Case presentation

**Figure 1:**
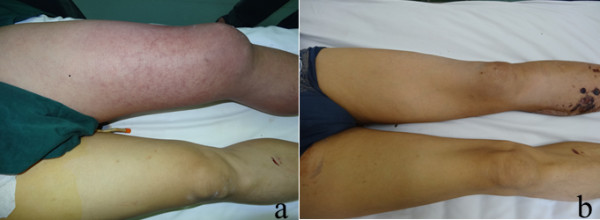
**The appearance of the left leg. a**. Initial appearance of the left leg, showing significant swelling and cyanosis. **b**. Appearance of the left leg 35 days after operation, at the time of the patient’s discharge.

**Figure 2:**
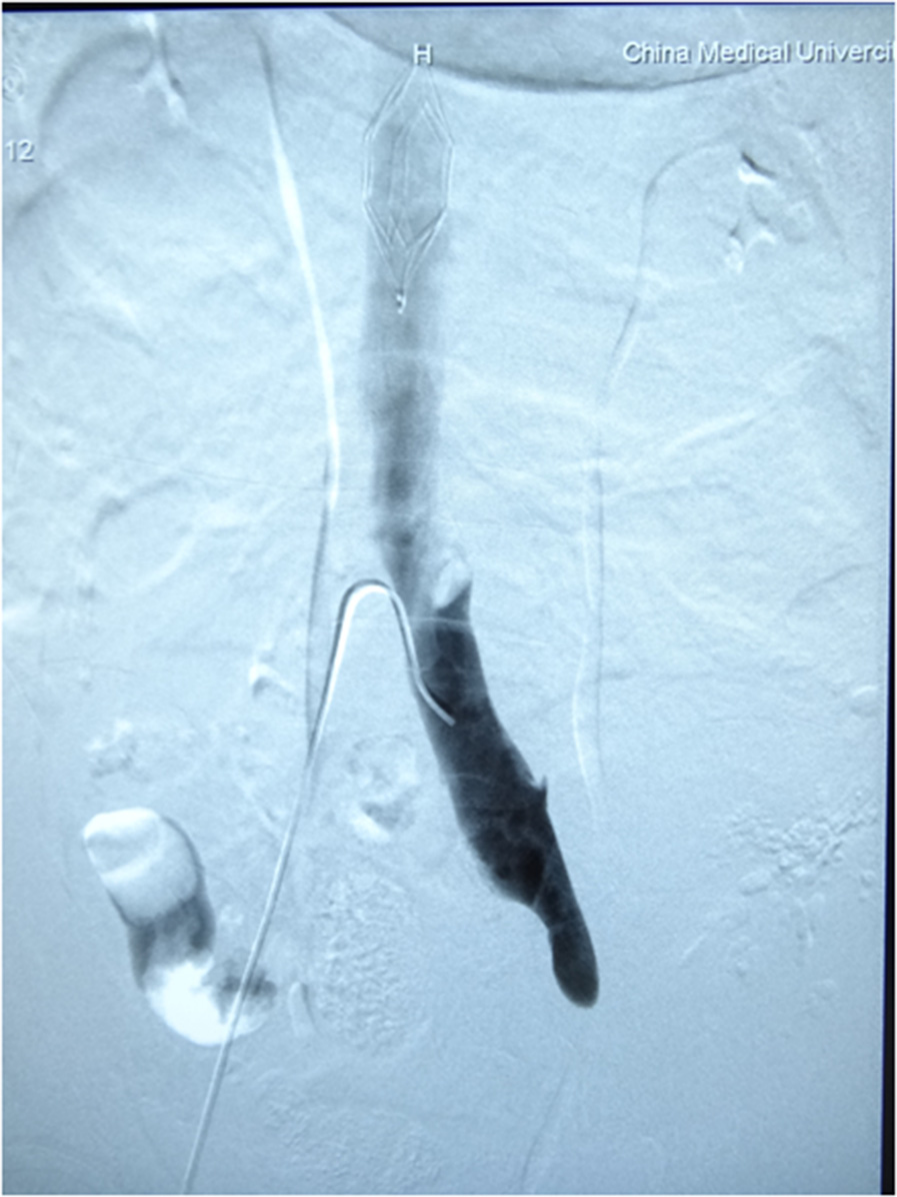
An OptEase® retrievable vena cava filter was placed in the inferior vena cava via the right femoral vein under digital subtraction angiography, and a venogram showed total occlusion of the left iliac vein.

**Figure 3:**
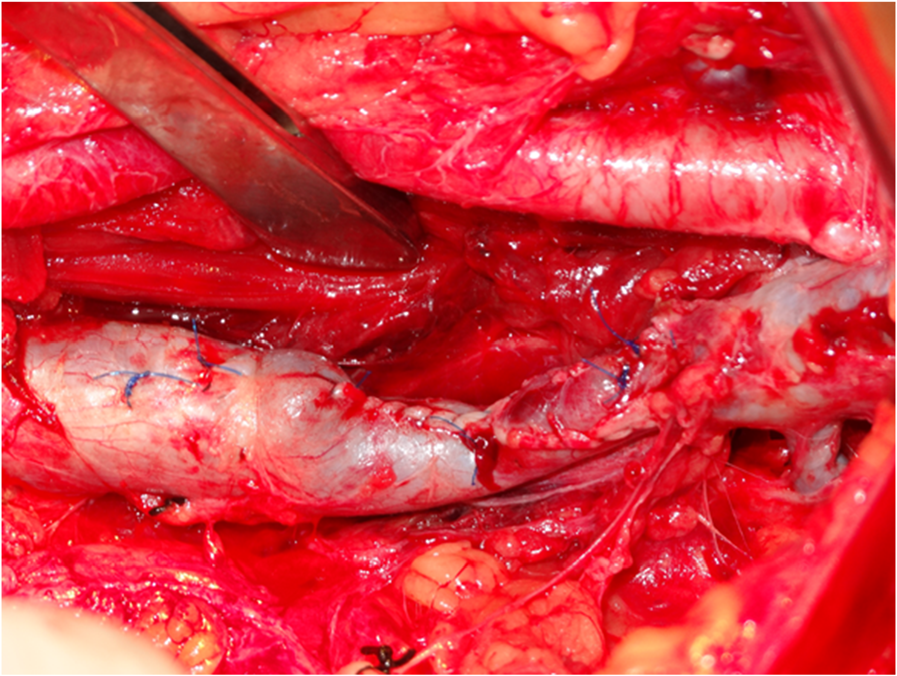
The left iliac vein, although narrower after repair, was patent.

A 49-year-old Chinese man sustained severe knife trauma and was referred to a local hospital. An exploratory laparotomy showed traumatic rupture of his left external iliac vein with massive intraperitoneal bleeding, but his iliac artery was intact. To rescue him, the proximal and distal parts of his injured external iliac vein were ligated (there was no vascular surgeon in the local hospital); however, his left leg quickly became severely swollen, cyanotic and pulseless. Two hours after the first operation, he was transferred to the emergency department of our university hospital with endotracheal tube intubation. On physical examination, he was tachycardic (142 beats per minute), hypotensive (86/50mmHg). His left lower extremity was markedly edematous, cyanotic, mottled (Figure 1a) and had no palpable pulse, but his right lower extremity was normal. He was diagnosed with posttraumatic PCD. Considering the high risk of pulmonary embolism during the next surgical procedure, an OptEase® retrievable vena cava filter (Cordis Corp., New Brunswick, NJ, USA) was placed in his inferior vena cava via his right femoral vein under digital subtraction angiography (Figure 2). His abdomen was immediately reopened while his left leg was also prepared for treatment.During the second emergent laparotomy, there was less blood in his pelvic cavity. No active bleeding was identified. His left iliac vein and artery were exposed and his entire iliac artery appeared normal and without rupture. His external iliac vein was ligated but not transected, connected only by the posterior wall, and the caudal part of his iliac vein was significantly dilated. By replacing the vascular clamp in the proximal and distal parts of the rupture, the ligations on both sides of the venous rupture were resolved. A wedge-shaped rupture was revealed, approximately 4cm in length, on the anterolateral side of his left external iliac vein. The edges of the venous rupture were smooth as a result of the knife trauma. Although the ligated parts of his iliac vein appeared congested, the lumen was smooth and contained no clot. The damaged vein was repaired with continuous 4-0 Prolene sutures (Ethicon, Inc, Somerville, NJ, USA) after the patient was systemically heparinized with 5000 units heparin. Before the sutures were finally ligated, the clamp at the cranial aspect was removed and the caudal clamp was left in place. After the backflow was clear of thrombotic debris, the clamp on his caudal iliac vein was removed and the clamp at the cranial aspect left in place. The left leg was gently massaged from calf to thigh; as a result, some thrombi in the caudal part of the vein were flushed out with the venous blood flow, and the sutures were finally ligated until the venous blood was free of thrombus. His iliac vein, although narrower after repair, was patent (Figure 3).

**Figure 4:**
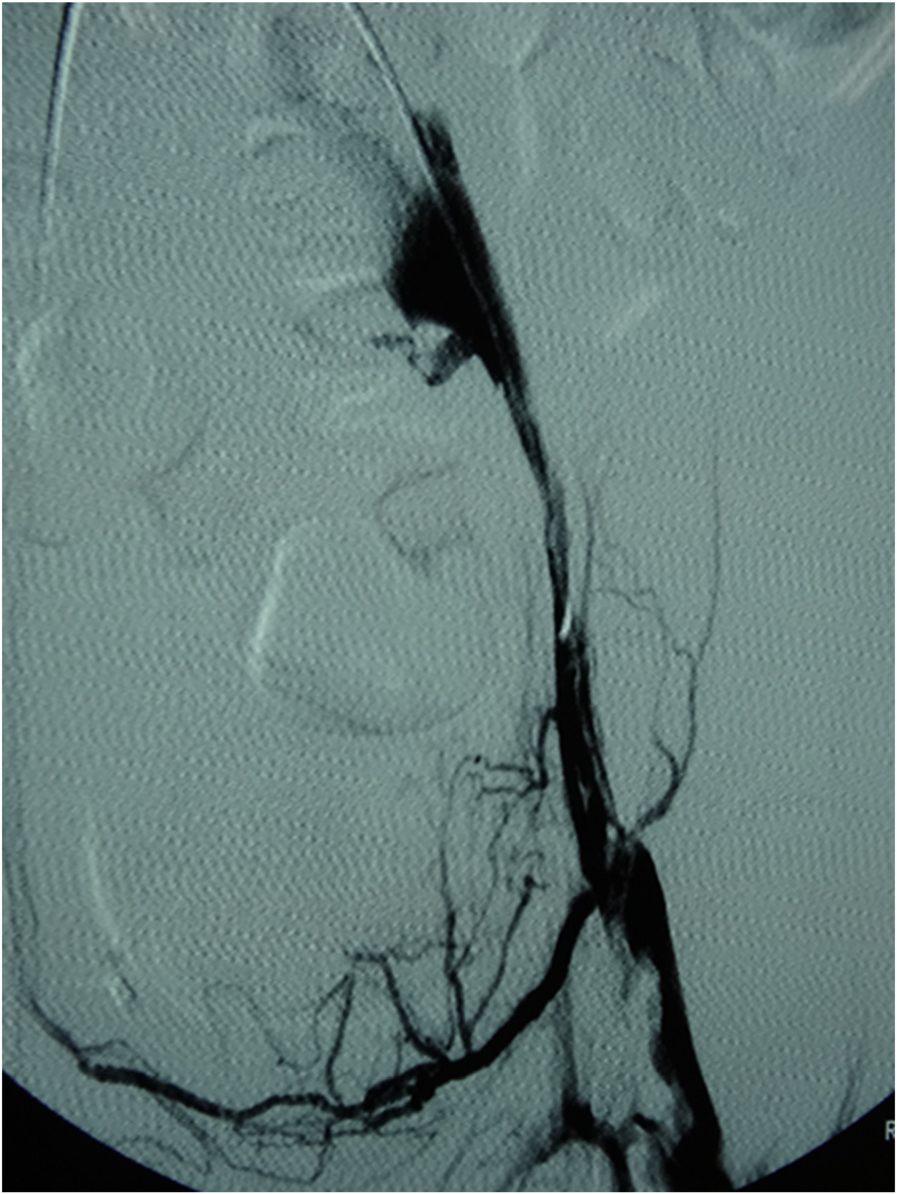
Final venogram showing a patent left external iliac vein with a small amount of residual thrombus.

After the operation, the patient was sent to the surgical intensive care unit with mechanical ventilation. Despite the fact that his left leg showed some signs of compartment syndrome with blisters in his calf, fasciotomy was not performed. He received systemic anticoagulation, initially with low-molecular-weight heparin, then with warfarin, titrating to an international normalized ratio (INR) between 2.0 and 2.5.Three days postoperatively, mechanical ventilation was discontinued and his signs and symptoms improved dramatically. Pulses in his left lower extremity became palpable on postoperative day 5, and the swelling and discoloration decreased. On postoperative day 30, he was taken off the OptEase® filter and the venogram showed a small amount of residual thrombus in his left external iliac vein (Figure 4). The inferior vena cava was patent, with no residual defects around the filter and no thrombus in the filter. He was discharged from the hospital in a stable condition, with full resolution of symptoms, on postoperative day 35 (Figure 1b). He was mostly asymptomatic at a recent 3-month follow-up visit except for slight edema in his left leg after walking. He continues on warfarin, maintaining an INR between 2.0 and 2.5, and wears elastic compression stockings.

## Discussion

PCD is a rare condition caused by complete venous occlusion that leads to impaired arterial flow [1-3]. It is usually a fulminant form of acute massive venous thrombosis, most often in the ileofemoral area, and the impaired drainage disorder results in tissue ischemia*.* This serious condition can lead to venous gangrene of the extremity or to compartment syndrome. PCD continues to have mortality rates of 25% to 40% and amputation rates of 20% to 50%, respectively [2,3,5]. Precipitating factors include malignancy, which is the most common cause; femoral vein catheterization; heparin-induced thrombocytopenia; antiphospholipid antibody syndrome; posttraumatic or postoperative conditions; heart failure; and pregnancy [6]. The diagnosis is usually made during clinical examination and confirmed by imaging studies.

There are few reports of PCD resulting from trauma [1,4]. Our case was one of posttraumatic PCD after emergency operation for iliac vein injury with massive intraperitoneal bleeding. Treatment of iliac vein injures remains extremely challenging even to an experienced trauma surgeon or vascular surgeon. Because the iliac veins are not mobile, an attempt to close a large defect may place the repair under tension, and repairing one small hole may become a repair of two larger ones. Hence, for a general surgeon without vascular or trauma experience, ligating the iliac vein without hesitation may be the best option when facing an injured iliac vein with massive bleeding [7]. In our case, the surgeons at the local hospital controlled the massive bleeding and rescued the patient by ligating the left iliac vein, thus creating a chance for further specialized vascular repair. However, as in our case, PCD resulting from ligation of an injured iliac vein should be considered in a severe trauma patient.

Traditionally, systemic anticoagulation with surgical thrombectomy has been the mainstay of treatment for PCD. More recently, systemic anticoagulation accompanied by pharmacomechanical catheter-directed deep venous thrombolysis has been shown to be the first-line treatment for patients with PCD [8-10]. In our case, however, the best way to treat PCD was to restore the venous drainage. As in surgical thrombectomy or catheter-directed deep venous thrombolysis, the risk of pulmonary embolism becomes a major concern during subsequent procedures. To prevent late caval thrombosis, it has been proven that use of the OptEase® retrievable vena cava filter provides safe and effective prophylaxis for thromboembolism during the procedures [11-14]. We did find thrombi in the caudal part of the ligated iliac vein, so the placement of a vena caval filter prior to therapy is advisable. Our experience of surgical thrombectomy showed that massaging the left leg from calf to thigh improved the evacuation of the thrombi with fairly good results. Our patient successfully underwent an emergency left iliac reconstruction for salvage of the limb following placement of a prophylactic inferior vena cava filter.

Because our patient did not sustain injury to the iliac arterial system, our case was similar to the spontaneous rupture of the iliac vein with PCD reported by Jazayeri *et al*. [15]. Luckily, we reconstructed the iliac vein with only a lateral repair. Had the patient not been saved with a lateral repair (that is, had the vein been transected or placed under tension, or if the patient had unstable vital signs), we would have considered alternative maneuvers. From the experience of Jazayeri *et al*. [15], a Palma-Dale bypass grafting procedure (crossover saphenous vein bypass grafting) with an arteriovenous fistula may be an alternative for restoration of venous drainage. Although Palma-Dale venous bypass in the acute phase of PCD remains controversial [1], it may be performed by an experienced vascular surgeon, not only for traumatic PCD, but also for PCD resulting from severe deep venous thrombosis when PCD fails to respond to any other treatment.

Our case shows that administration of warfarin with close postoperative follow-up is effective in preventing thrombosis. As in the treatment of deep venous thrombosis, postoperative anticoagulation treatment is mandatory. Leg elevation, oral anticoagulation therapy, and elastic compression stockings may be necessary for several months postoperatively.

## Conclusions

In summary, we have presented a case of posttraumatic PCD resulting from an injured iliac vein treated at a small local hospital. If specialized surgery is not available, prompt transfer to a tertiary-care hospital capable of providing appropriated care is mandatory. Prompt reconstruction of the injured iliac vein to restore the venous drainage and direct evacuation of the venous thrombi provided a timely and effective treatment of posttraumatic PCD with impending gangrene.

## Consent

Written informed consent was obtained from the patient for publication of this case report and any accompanying images. A copy of the written consent is available for review by the Editor-in-Chief of this journal.

## Abbreviations

INR: International normalized ratio; PCD: Phlegmasia cerulea dolens.

## Competing interests

The authors declare that they have no competing interests.

## Authors’ contributions

HH, YC, and CW joined the emergent operation and our patient was admitted to our hospital under the care of CY. JZ carefully searched the literature on PubMed; ZD and SX reviewed the manuscript and gave some instruction and advice. HH and YC were major contributors in writing the manuscript. All authors read and approved the final manuscript.
